# Combination Trimodality Therapy Using Vismodegib for Basal Cell Carcinoma of the Face

**DOI:** 10.1155/2015/827608

**Published:** 2015-10-04

**Authors:** Alec M. Block, Fiori Alite, Aidnag Z. Diaz, Richard W. Borrowdale, Joseph I. Clark, Mehee Choi

**Affiliations:** ^1^Department of Radiation Oncology, Stritch School of Medicine, Loyola University Medical Center, 2160 S. First Avenue, Maywood, IL 60153, USA; ^2^Department of Radiation Oncology, Rush University Medical Center, 500 S. Paulina Street, Ground Floor, Chicago, IL 60612, USA; ^3^Department of Otolaryngology Head and Neck Surgery, Stritch School of Medicine, Loyola University Medical Center, 2160 S. First Avenue, Maywood, IL 60153, USA; ^4^Department of Medicine, Division of Hematology/Oncology, Stritch School of Medicine, Loyola University Medical Center, 2160 S. First Avenue, Maywood, IL 60153, USA

## Abstract

*Background*. For large basal cell carcinomas (BCCs) of the head and neck, definitive surgery often requires extensive resection and reconstruction that may result in prolonged recovery and limited cosmesis. Vismodegib, a small-molecule inhibitor of the hedgehog pathway, is approved for advanced and metastatic BCCs. We present a case of advanced BCC treated with combination of vismodegib, radiotherapy, and local excision resulting in excellent response and cosmesis. *Case Presentation*. A 64-year-old gentleman presented with a 5-year history of a 7 cm enlarging right cheek mass, with extensive vascularization, central ulceration, and skin, soft tissue, and buccal mucosa involvement. Biopsy revealed BCC, nodular type. Up-front surgical option involved a large resection and reconstruction. After multidisciplinary discussion, we recommended and he opted for combined modality of vismodegib, radiotherapy, and local excision. The patient tolerated vismodegib well and his right cheek lesion decreased significantly in size. He was then treated with radiotherapy followed by local excision that revealed only focal residual BCC. Currently, he is without evidence of disease and has excellent cosmesis. *Conclusions*. We report a case of locally advanced BCC treated with trimodality therapy with vismodegib, radiotherapy, and local excision, resulting in excellent outcome and facial cosmesis, without requiring extensive resection or reconstructive surgery.

## 1. Introduction

For small, early stage, localized basal cell carcinoma (BCC) of the head and neck, primary surgical resection or primary radiation therapy is the mainstay of treatment [1, 2]. For more advanced and metastatic cases, however, the role of definitive surgery or radiation therapy alone is limited. Vismodegib, a small molecule inhibitor of the hedgehog pathway which is upregulated and causes uncontrolled proliferation of basal cells in BCC, has previously been shown to elicit response rates ranging from approximately 30% to 60% in advanced and metastatic cases, with a well-tolerated side effect profile [3–6]. Moreover, in a landmark phase 2 study, biopsies of patients with locally advanced BCC treated with vismodegib alone revealed a complete pathologic response rate of 54% [4]. Based on these results, vismodegib became the first hedgehog signaling pathway targeted agent to gain US Food and Drug Administration (FDA) approval on January 30, 2012.

Several previous cases using vismodegib with combination therapy have been reported. In one such report, radiation therapy was used to treat squamous cell carcinoma of the skin while vismodegib was concurrently used for treatment of multiple BCC lesions [7]. In this single case, the authors demonstrated that radiation therapy for squamous cell carcinoma could be delivered safely and effectively at the same time as treatment with vismodegib [7]. Similarly, 2 cases were reported in which patients had an excellent clinical and radiographic response following completion of combination of vismodegib with concurrent radiation therapy for recurrent, locally advanced BCC [8]. For more advanced cases, potential use of vismodegib may include neoadjuvant treatment prior to a planned surgery, thus allowing for a smaller resection and subsequent reconstruction. A case utilizing this treatment paradigm has been reported with promising results [9]. Although vismodegib in combination with surgery alone or radiation therapy alone has been reported, to our knowledge, there have been no reports using all three modalities. Therefore, we present a case of locally advanced BCC of the face treated with vismodegib, radiation therapy, and ultimately local excision, without requiring a major resection or reconstruction and resulting in excellent function and cosmesis.

## 2. Case Report

**Figure 1:**
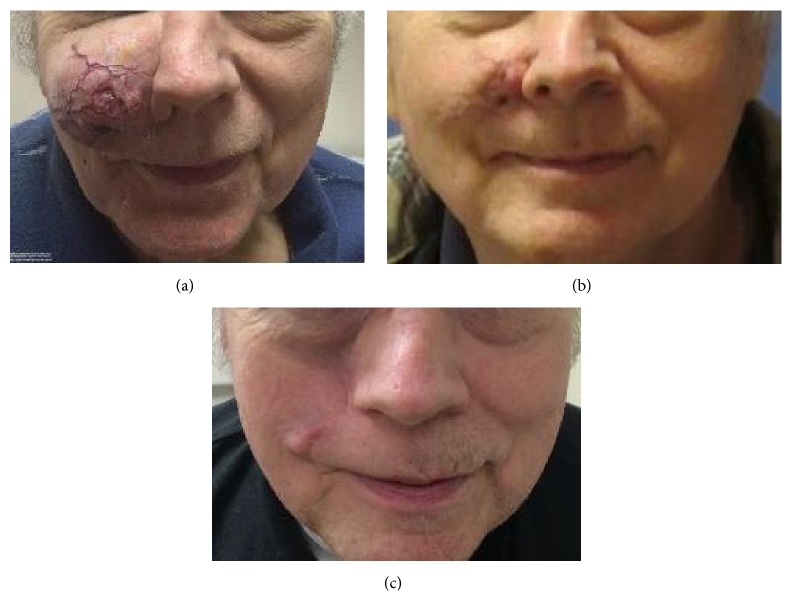
Clinical images. Photographs of the patient at the time of initial presentation (a), after 4 months of vismodegib therapy (b), and at first follow-up, 2 months after completion of trimodality therapy (c).

A 64-year-old gentleman presented with a 5-year history of an enlarging right cheek mass. He reported that the lesion was not bothersome at first but that it had been growing slowly over time. He presented because the mass had grown so much in size that it was obscuring his inferior visual field to the point that he was unable to see beneath his cheek on the right side. He denied numbness or tingling of the face, facial pain, weight loss, or difficulty with chewing. He had no other bumps or masses and no other complaints. His past medical history was significant for hypertension, hyperlipidemia, coronary artery disease with 3 myocardial infarctions and percutaneous coronary artery stenting, and an inguinal hernia repair. He walked with crutches for a left ankle fracture that he sustained as a youth. He was a previous cigar smoker but denied alcohol or illicit drug use. His father had BCC of the face, and his sister had breast cancer. Physical examination was significant for a 7 cm by 5 cm right cheek mass with extensive vascularization and central ulceration (see Figure 1(a)). The lesion involved the skin and soft tissues of the face and extended to the buccal mucosa of the right cheek but was mobile and did not appear fixed to the maxilla. He had numbness on the right side of his face in the distribution of cranial nerve V2. There was no palpable facial or cervical neck lymphadenopathy.

**Figure 2:**
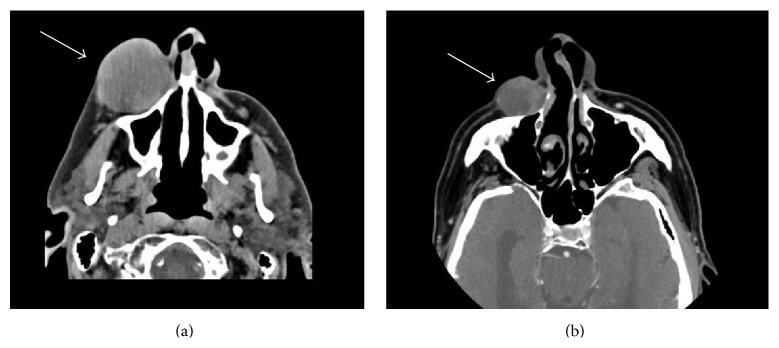
Radiographic images. Radiographic findings of facial bone computed tomography (CT) scan at time of initial presentation (a) and following 4 months of vismodegib therapy (b).

Noncontrast facial bone computed tomography (CT) scan revealed a mass-like subcutaneous lesion abutting the anterior aspect of the right maxilla, maxillary sinus, and inferior orbital rim and base of nasal bone, measuring about 5.5 cm in length by 5 cm in width by 4.5 cm in anterior to posterior dimension (see Figure 2(a)). No definite bone erosion or remodeling was demonstrated. No enlarged lymph nodes were evident in the field of view. Posterior-anterior and lateral 2-view chest X-ray was benign. Ultrasound guided fine needle aspiration of the mass revealed BCC of nodular type, staged as clinical T2N0M0, Stage II.

His case was discussed at multidisciplinary tumor board and the consensus was that up-front surgical monotherapy would involve a large full thickness resection of the skin of the face, likely with a frozen section of the infraorbital nerve, a full thickness resection through the cheek including removal of the buccal mucosa, and a radial forearm free flap reconstruction. Given his major medical comorbidity of coronary artery disease with multiple previous myocardial infarctions, there was concern that he may be medically unfit for such an extensive surgical procedure. Moreover, the patient was concerned about his postoperative recovery period and ultimate facial cosmesis following such an approach. Alternatively, he was offered combined modality therapy with once daily oral vismodegib 150 mg, followed by definitive radiation therapy once the response to vismodegib had either dramatically slowed or plateaued, reserving surgery for salvage. He was agreeable to this plan and vismodegib was initiated.

Overall, he tolerated the vismodegib very well and denied any muscle spasms, hair loss, weight loss, fatigue, nausea, decrease in appetite, or diarrhea. His only new complaint after 4 months of therapy was a minor decrease in taste which did not affect his appetite, ability to eat, or weight. Within 2 weeks of taking vismodegib, he noticed a decrease in the size of his lesion, and on physical exam it decreased to 6 cm by 4 cm. At 6-week follow-up, the lesion was 5 cm by 3 cm, and at 10-week follow-up it measured 4 cm by 3 cm. After approximately 14 weeks of vismodegib, the rate of reduction in the size of the lesion decreased, so the decision was made to proceed with definitive radiation therapy. The patient was maintained on vismodegib until the initiation of radiation therapy, resulting in approximately 4 months of drug therapy in total that resulted in an objective clinical response of greater than 50% reduction in the size of the lesion (see Figure 1(b)). Prior to starting radiation therapy, he underwent repeat facial bone CT scan which revealed that the lesion had decreased in size to 2.7 cm in largest dimension (see Figure 2(b)).

**Figure 3:**
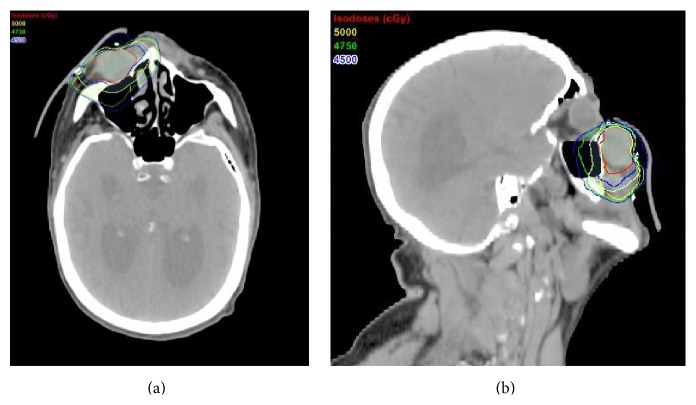
Radiation therapy treatment plan. Axial (a) and sagittal (b) views of the radiation therapy treatment plan with target volumes and representative dose distributions: gross tumor volume (GTV) in red, clinical target volume (CTV) in blue, planning target volume (PTV) in cyan, and 45 Gy (blue), 47.5 Gy (light green), and 50 Gy (yellow) isodose lines.

He then underwent CT simulation, in which he was positioned supine with a head and neck thermoplastic immobilization mask with a radioopaque wire placed around his residual lesion. His radiation therapy prescription was 50 Gy in 20 fractions of 2.5 Gy per fraction to the gross residual lesion (gross tumor volume, GTV) plus margin accounting for both local microscopic spread (clinical target volume, CTV) and interfraction setup variability (planning target volume, PTV) delivered daily, Monday through Friday, for a total of 4 weeks. He was treated with a 0.5 cm daily skin bolus using 3-dimensional conformal radiation therapy (3DCRT) with a 4-field technique involving right anterior oblique, left anterior oblique, anterior superior oblique, and anterior inferior oblique field arrangements.  Figure 3 shows representative views and dose distributions of the radiation therapy plan on his CT simulation scan.  Table 1 relates target volumes to dose coverage and also shows representative doses to nearby critical structures. He tolerated radiation therapy very well with the expected toxicities of grade 1 fatigue that did not limit his daily activities and grade 2 moist skin desquamation in the area of the nasolabial fold that was improved with over-the-counter moisturizer and topical antibiotic cream.

At 3-month follow-up after the completion of radiation therapy, it was noted that he had a persistent 1.5 cm firm nodule in the right nasal-alar groove with overlying vasculature. Based on physical exam alone, it was difficult to determine if this nodule was scarring versus residual malignancy. Due to its firmness, size, and location, it was not amenable to fine needle aspiration, so the patient was taken for wide local excision, 2.5 cm by 5 cm, with intermediate closure. During the procedure, an elliptical incision was performed in the right cheek and the BCC in the right nasolabial fold was excised, resulting in a 2.5 cm by 5 cm defect which was then closed. He did not require reconstruction. Pathology from the wide local excision revealed only focal residual BCC with negative surgical margins. Overall, he tolerated the treatment very well and complained only of minor skin tightness and nasal congestion following his surgery. At 2-month follow-up after his surgery, he was doing very well, was clinically without evidence of disease, and had excellent facial cosmesis and functional capacity (see Figure 1(c)).

## 3. Conclusions

Mutations in hedgehog pathway genes have been implicated in several malignancies, including BCC [3–6]. Vismodegib, a first in class small molecule inhibitor of smoothened homologue, a key component of the hedgehog pathway, has been shown to be effective in the treatment of advanced and metastatic BCC [3–5]. In the setting of such promising results, particularly in a disease in which other systemic options are limited, there will be continued interest in utilizing vismodegib in combination with local therapy, surgery, and/or radiation therapy, for treatment of locally advanced nonmetastatic BCC, thus allowing for a smaller surgical procedure or smaller radiation therapy field.

Cases of combination of vismodegib with surgery alone or radiation therapy alone have been reported [7–9]. To our knowledge, this is the first case in which trimodality therapy—vismodegib, radiation therapy, and outpatient local excision—was used to treat locally advanced facial BCC that otherwise would have required a larger surgical resection, free flap reconstruction, overnight hospitalization, and overall significantly longer recovery time. Particularly in our patient's case, in which there was concern that he might be medically unfit for such a large procedure because of his significant cardiac comorbidities, this trimodality approach presented an excellent option for oncologic control while maintaining facial cosmesis. Moreover, this report offers an example of the tolerability and safety of such an approach. Other than mild dysgeusia associated with vismodegib, he did not experience other previously reported toxicities, such as hair loss, muscle spasms, weight loss, nausea, decreased appetite, and diarrhea [4]. Following vismodegib, his radiation therapy was also well tolerated, and he did not have any perioperative surgical complications even after undergoing treatment with both vismodegib and radiation therapy. Similar to all patients treated with vismodegib, he will continue to be monitored closely, particularly for emergence of secondary squamous cell carcinoma lesions that may arise as sequelae from small molecule hedgehog pathway inhibitor therapy, which has been well documented previously [10–12].

Given the promising results of vismodegib in advanced and metastatic BCC, there will undoubtedly be growing popularity in a similar approach to that used in our patient in which vismodegib is combined with local therapy for locally advanced BCC. Although there are now several reported cases using vismodegib with surgery or radiation therapy, the most appropriate timing and sequencing of these modalities in order to provide the best outcomes with the least toxicity are widely unknown. A recent clinical trial, NCT01543581: Placebo-controlled, Double Blind Study to Assess Efficacy and Safety of Oral Vismodegib for the Treatment of BCC Preceding Excision by Mohs Micrographic Surgery (MMS), used vismodegib as neoadjuvant therapy prior to surgical resection in order to assess the efficacy and safety of vismodegib compared to placebo in the oral adjunctive presurgical treatment of BCC [13]. This study has closed to accrual and results are pending. Similarly, another trial, NCT01201915: A Phase II, Multicenter, Open-label, Three-cohort Trial Evaluating the Efficacy and Safety of Vismodegib (GDC-0449) in Operable BCC, is evaluating the pathologic complete response rate in patients with operable BCC undergoing vismodegib therapy [14]. This study has also closed to accrual and results are pending. Finally, another trial, NCT01835626: A Phase II Study of Radiation Therapy and Vismodegib, for the Treatment of Locally Advanced BCC of the Head and Neck, is currently open to accrual and aims to assess the safety and tolerability of combined radiation therapy and concurrent vismodegib [15].

We await the results of these trials, which will shed more light on the role of vismodegib in combination with local therapy for the treatment of locally advanced BCC. In our patient, we found that combination of vismodegib, radiation therapy, and outpatient local excision for locally advanced BCC of the face was safe and well tolerated, allowed for a less extensive recovery that otherwise may have been unfavorable in a patient with severe cardiac comorbidities, and resulted in excellent oncologic control while maintaining function and cosmesis. Long-term follow-up of our patient is needed to determine long-term response to treatment.

## Figures and Tables

**Table 1:** Dose-volume histogram data.

Dosimetric characteristic	Achieved
GTV, *D*_95%_	100%
CTV, *D*_95%_	99%
PTV, *D*_95%_	97%
Right eye, *D*_max⁡_	44 Gy
Right eye, mean	27 Gy
Right lacrimal gland, *D*_max⁡_	31 Gy
Right lacrimal gland, mean	24 Gy
Right optic nerve, *D*_max⁡_	30 Gy
Right optic nerve, mean	20 Gy

GTV: gross tumor volume; *D*_*x*%_: percent of prescribed dose delivered to *x*% of volume; *D*_max⁡_: maximum dose; Gy: Gray; CTV: clinical target volume; PTV: planning target volume.

## Abbreviations

BCC: Basal cell carcinoma
CT: Computed tomography
GTV: Gross tumor volume
CTV: Clinical target volume
PTV: Planning target volume
3DCRT: 3-Dimensional conformal radiation therapy
MMS: Mohs micrographic surgery.
